# Freeform Mirror Design Based on Zonal Energy Mapping

**DOI:** 10.3390/ma19173682

**Published:** 2026-08-30

**Authors:** Fei Wang, Xin Zhang, Yunhai Tang, Yue He, Baohua Chen

**Affiliations:** 1 School of Computing Science and Artificial Intelligence, Suzhou City University, Suzhou 215104, China; wangfei@szcu.edu.cn; 2 Suzhou Key Laboratory of Multi-Modal Data Fusion and Intelligent Healthcare, Suzhou City University, Suzhou 215104, China; 3 Suzhou Institute of Biomedical Engineering and Technology, Chinese Academy of Sciences, Suzhou 215163, China; 4 Key Laboratory of Efficient Low-Carbon Energy Conversion and Utilization of Jiangsu Provincial Higher Education Institutions, School of Physical Science and Technology, Suzhou University of Science and Technology, Suzhou 215009, China; 18206224192@163.com (Y.H.); chenbaohua@usts.edu.cn (B.C.); 5 Graduate Practice Station in Soochow Mason Optics Co., Ltd., Suzhou 215127, China

**Keywords:** beam shaping, freeform mirror, energy mapping, M-shaped irradiance profile, high-power laser processing

## Abstract

In laser phase-transformation hardening and laser cladding processes, the inherent thermodynamic limitations of conventional intensity distributions severely restrict the uniformity of metallurgical reaction. Although programmable beam shaping devices offer flexibility in profile reconstruction, their transmissive structure results in a low laser-induced damage threshold (LIDT), making them unsuitable for long-term stable operation at kilowatt-level power. This study proposes a reflective freeform mirror design method based on the principle of zonal energy mapping. The method constructs energy mapping relations that correlate the irradiance distribution in every sub-region of the incident Gaussian beam with the desired M-shaped irradiance profile. Relying on such mappings, the local surface generatrices of all sub-regions are solved separately; these generatrices are subsequently assembled to yield an integrated mirror surface. Optical performance was verified through Zemax non-sequential ray tracing simulations. The mirror was precisely machined using single-point diamond turning (SPDT). The experimental results show that the measured intensity distribution on the observation screen exhibits a typical M-shaped profile, with a peak-to-valley ratio of 1.28, which is in good agreement with the design target. This method provides a technically feasible and engineering-robust solution for complex thermal flux control requirements in high-power laser material processing.

## 1. Introduction

Laser transformation hardening (LTH) and laser cladding have emerged as pivotal surface engineering technologies for enhancing the wear and fatigue resistance of metallic components without compromising bulk properties [1,2,3]. However, the attainment of a uniform case depth remains fundamentally constrained by the mismatch between the incident laser irradiance profile and the transient thermal boundary conditions. Conventional TEM_00_ Gaussian modes exhibit a pronounced central energy density, which inevitably induces martensite overheating or even melting at the scan center, while leaving the peripheral regions insufficiently austenitized [4,5,6]. Conversely, homogenized top-hat profiles, though mitigating central peaks, fail to counteract lateral heat dissipation into the colder substrate. This thermodynamic asymmetry leads to a characteristic “crescent-shaped” hardness trajectory and poor process repeatability [1].

To tailor the spatial energy distribution, programmable devices such as liquid crystal spatial light modulators (LC-SLMs) [7,8,9] and diffractive optical elements (DOEs) have been employed to generate complex profiles, including the theoretically optimal M-shaped irradiance distribution [10,11,12].

The laser-induced damage threshold (LIDT) of liquid crystal spatial light modulators (LC-SLMs) is only 155 W/cm^2^ [7]; diffraction optical elements (DOEs), due to material absorption and thermal accumulation effects from microstructures, typically have an LIDT below 10^3^ W/cm^2^, limiting their use primarily to low-power industrial applications such as laser imaging, micro/nanofabrication, and plastic welding [11]. In contrast, reflective integrators offer excellent mechanical robustness and scalable active water-cooling thermal management. After being coated with high-performance dielectric high-reflectivity films, their measured LIDT can reach up to 6.72 × 10^8^ W/cm^2^ [13], representing an improvement of over six orders of magnitude compared to LC-SLMs. As a result, reflective integrators have become the mainstream solution in high-power laser industrial applications. However, traditional mirror designs rely solely on simple beam splitting and superposition, thus inherently facing difficulties in achieving customized gradient control of light intensity distribution [14,15,16].

To address this technical gap, this study proposes a freeform mirror design method that combines adherence to the law of energy conservation with the intensity superposition approach. Unlike traditional beam-splitting methods, our approach establishes a continuous and one-to-one energy mapping relationship between the energy distribution in each subregion of the incident Gaussian beam and the corresponding local intensity target. Based on this mapping relationship and the law of reflection, we achieved precise control over the peak-to-valley ratio of the intensity distribution. This paper presents a geometric optics mapping model for M-shaped beam generation. Using non-sequential ray tracing, we quantitatively simulated beam shaping and achieved M-shaped intensity profiles. We fabricated mirror prototypes and experimentally confirmed their stable, precise conversion of Gaussian input into the target M-shaped profile—enabling intensity control in laser material processing.

## 2. Design of the Energy-Conserving Freeform Mirror

### 2.1. Theoretical Framework and Energy Mapping Principle

Unlike conventional integral mirrors that rely on beam splitting and superposition, the proposed freeform mirror design takes the law of energy conservation as the core constraint, establishing a quantitative mapping relationship between the incident Gaussian irradiance and the target output irradiance. This enables an inverse design workflow from the desired energy distribution to the mirror geometry, breaking the inherent limitation of traditional integrators that only produce uniform output profiles.

**Figure 1:**
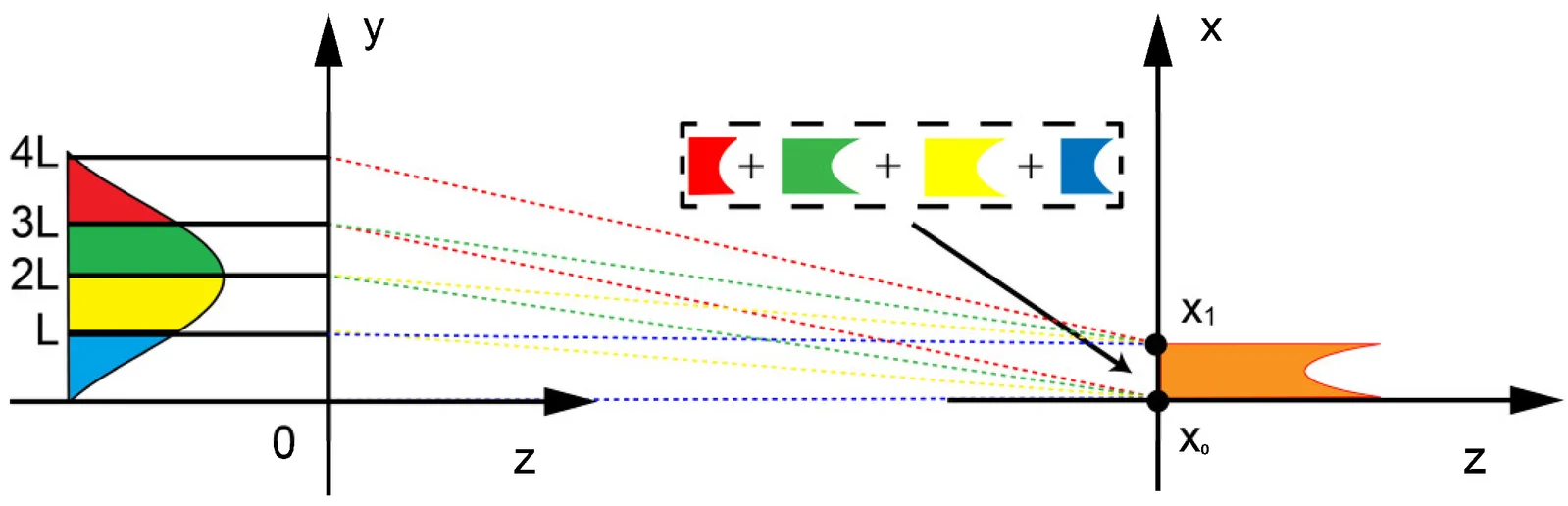
Schematic of the energy mapping relationship between the incident Gaussian beam and the target plane.

As illustrated in Figure 1, a Cartesian coordinate system is defined with the incident beam propagating along the +*z* direction. The incident beam is assumed to be a TEM_00_ Gaussian beam with a waist radius of *w*_0_, whose irradiance distribution along the *y*-axis is given by:

(1) $$I_{\mathrm{in}\;}(y)=I_{0}\exp\left(-\frac{2y^{2}}{w_{0}^{2}}\right),$$

where $I_{0}$ is the peak irradiance at the beam center, and *y* denotes the coordinate along the direction of the incident cross-section.

The freeform mirror in this study is made of high thermal conductivity metal and integrates an internal microchannel liquid cooling system. Under steady-state laser irradiation, the measured temperature gradient on the mirror surface is low, allowing spatially non-uniform thermal deformation to be neglected. For an ideal, uniform mirror surface, the refractive index of a given material layer remains constant across the surface; accordingly, its optical reflection characteristics—including reflectivity and phase response—also exhibit spatial uniformity.

The beam intensity distribution along the *y*-axis is divided into *N* equal subregions, each of which projects onto the same position on the *x*-axis in the target plane, thereby establishing a deterministic mapping relationship between the *y*-coordinate of the incident beam and the *x*-coordinate of the target spot.

To enable numerical implementation, the incident beam aperture along the *y*-axis is discretized into equal segments with a segment length of $L=L_{\mathrm{in}\;}/N$, where $L_{\mathrm{in}}$ is the effective incident beam aperture. The *n*-th segment spans the interval $\left|y_{n-1}-y_{n}\right|=L$, with $y_{n}=nL,\;(n=1,2,\ldots ,N)$.

Let the intensity distribution of the target spot in the *x*-direction be $I_{\mathrm{out}}(x)$. $I_{\mathrm{out}}(x)$ is the predefined target irradiance profile (e.g., uniform, M-shaped, or other customized distributions for laser thermal processing). This function is defined over the entire target domain and satisfies the normalization constraint, with the integral evaluated over the complete target region.

(2) $$\int_{x_{0}}^{x_{1}}I_{\mathrm{out}}(x)dx=1,$$

where *x*_0_ and *x*_1_ represent the spatial boundary coordinates of the target spot in the *x*-direction, i.e., the left and right endpoints of its defined interval.

The output spot intensity distribution corresponding to the *n*-th subregion of the incident beam is denoted as $I_{\mathrm{out},n}(x)$, defined along the *x*-direction on the target plane, representing the local contribution of this subregion to the final synthesized intensity.

(3) $$I_{\mathrm{out},n}(x)=I_{\mathrm{out}}(x)\int_{y_{n-1}}^{y_{n}}I_{\mathrm{in}\;}(y^{\prime })dy^{\prime }.$$

The total output intensity distribution along the target *x*-axis satisfies the superposition relation as follows:

(4) $$I_{\mathrm{total},\mathrm{out}}(x)=\sum_{n=1}^{N}I_{\mathrm{out},n}(x).$$

According to the law of energy conservation, the optical energy carried by each incident sub-region must be fully coupled into the same overlapping interval on the target plane’s *x*-axis. The constraint is mathematically expressed as:

(5) $$\int_{y_{n-1}}^{y_{n}}I_{\mathrm{in}\;}(y^{\prime })dy^{\prime }=\int_{x_{0}}^{x_{1}}I_{\mathrm{out},n\;}(x^{\prime })dx^{\prime },$$

and $\left|x_{0},x_{1}\right|$ is the target interval. The origin of the target *x*-axis is aligned with the left edge of the desired spot, such that *x*_0_ = 0 marks the starting position of the output profile. A sufficient condition for Equation (5) is derived from differential energy conservation [17], i.e.,

(6) $$I_{\mathrm{in}}(y)dy=\sigma I_{\mathrm{out},n}(x)dx,$$

where *y* is the vertical coordinate in the input plane and *x* is the horizontal coordinate in the output plane, $\sigma =(y_{n}-y_{n-1})/(x_{1}-x_{0})$. To ensure that the cumulative energy at position *x* in the output equals exactly the cumulative energy at position *y* in the input, a nonlinear mapping of the output coordinates is required:

(7) x=M(y)=m(y)·y,

where *m*(*y*) is a differentiable scaling function. Equations (6) and (7) can be combined to yield:

(8) $$d\left[m(y)\cdot y\right]=\frac{I_{\mathrm{in}}(y)}{\sigma I_{\mathrm{out},n}\left[m(y)\cdot y\right]}dy.$$

Given that the incident irradiance profile is divided into *n* subregions along the *y*-axis, the *n*-th subregion starts at *y*(0) = (*n*−1)*L*, integrating the above expression yields:

(9) $$y\cdot m(y)-y_{n}(0)\cdot m\left[y_{n}(0)\right]=\int_{y_{n}(0)}^{y}\frac{I_{\mathrm{in}}(u)}{\sigma I_{\mathrm{out},n}\left[m(u)\cdot u\right]}du,$$

Since the intensity of the outgoing light spot equals zero at *x*_0_ = 0, i.e., $y_{n}(0)\cdot m\left[y_{n}(0)\right]=0$, we can obtain:

(10) $$m(y)=\frac{1}{y}\int_{y_{n}(0)}^{y}\frac{I_{\mathrm{in}}(u)}{\sigma I_{\mathrm{out},n}[m(u)\cdot u]}du.$$

Solving for the coordinate mapping relation *x* = *M*(*y*) that satisfies cumulative energy conservation between the incident intensity distribution $I_{\mathrm{in}}(y)$ and the output intensity distribution $I_{\mathrm{out},n}(x)$. This mapping directly determines the local curvature and normal deflection angle required for the integrating mirror, thereby providing a theoretical basis for the quantitative design of the mirror’s optical surface profile.

### 2.2. Design of Free-Form Surface Reflectors

**Figure 2:**
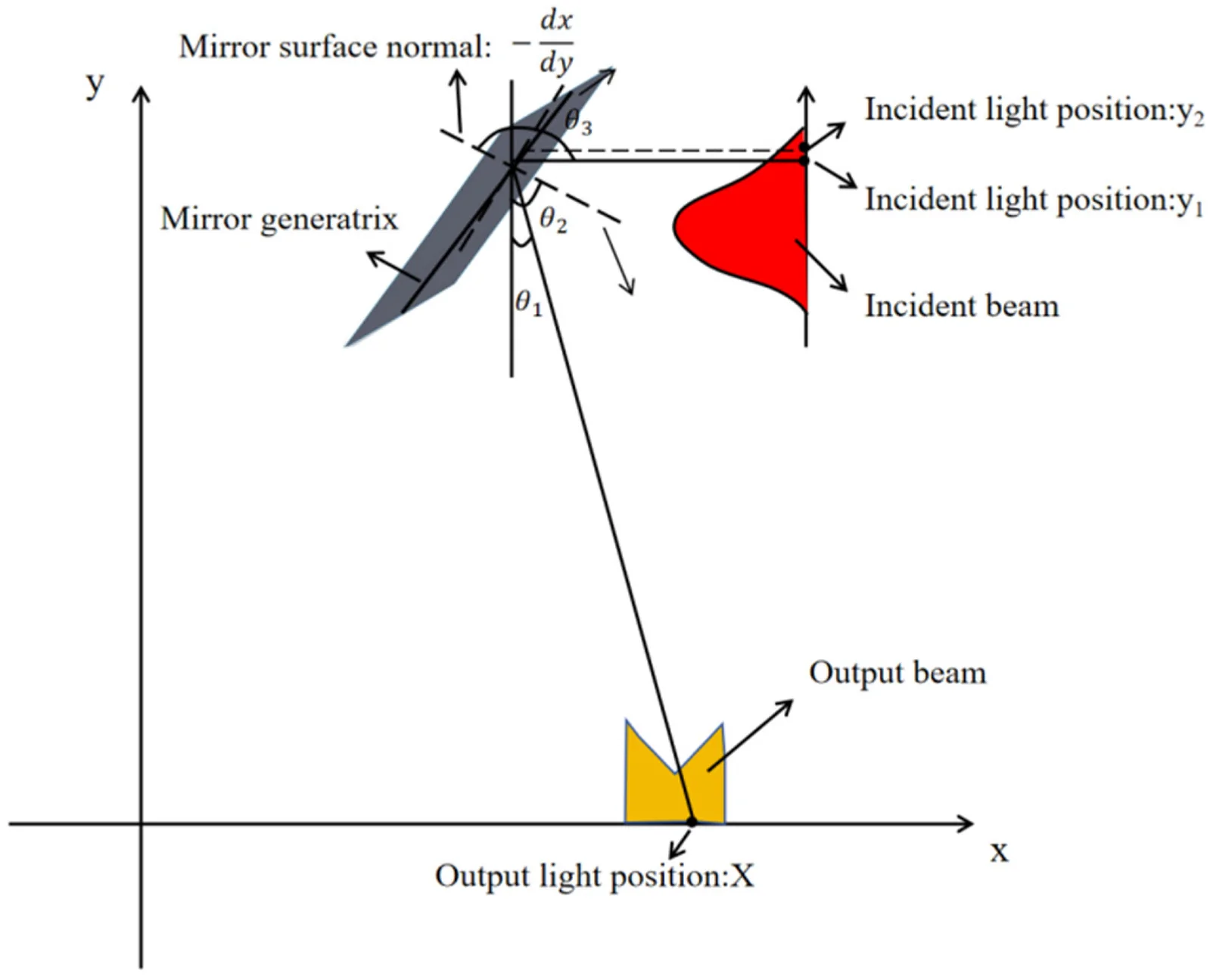
Schematic illustration of the coordinate-mapping-based freeform mirror design principle.

Based on the aforementioned coordinate mapping between output and input positions, the geometric profile of the integrating mirror can be determined pointwise. As illustrated in Figure 2, a collimated beam incident along the *y*-direction at position *y* is reflected at a mirror surface point (*x*_m_, *y*_m_) and precisely directed onto the output position x=m(y)·y on the target plane. The lateral coordinate *x*_m_ and vertical height *y*_m_ of this reflection point are jointly constrained by the law of reflection and the mapping relation, thereby uniquely determining the local surface normal vector and curvature required at that location.

To facilitate the numerical implementation, the incident light intensity is discretized into equal segments along the mirror generatrix. According to the energy conservation law, the optical energy carried by each incident segment is fully transferred to an overlapping region on the target plane. This bijective mapping relationship establishes the foundation for deriving the continuous surface generatrix.

Let *x* denote the *x*-coordinate of the output plane corresponding to the incident beam coordinate *y* of the *n*-th subsegment, which reads:

(11) $$x=x_{0}+m(y)\cdot y,$$

where *x*_0_ is the displacement constant selected for the working surface, and (n−1)L<y<nL.

According to the law of reflection and the geometric relationship shown in Figure 2, we obtain *y*_m_ = *y*; from this, a series of equations can be derived.

(12) $$tan\theta_{1}=\frac{x-x_{m}}{y},$$

(13) $$\theta_{2}=\frac{\pi }{4}+\frac{\theta_{1}}{2},$$

(14) $$\theta_{3}=\frac{3}{4}\pi +\frac{\theta_{1}}{2},$$

(15) $$-\frac{dx_{m}}{dy_{m}}=\tan(\frac{3}{4}\pi +\frac{\theta_{1}}{2}).$$

From the relationships among trigonometric functions, we can derive:

(16) $$\frac{dy_{m}}{dx_{m}}=\frac{1+\tan\frac{\theta_{1}}{2}}{1-\tan\frac{\theta_{1}}{2}}.$$

Let $\tan\theta_{1}/2=t$; then we have:

(17) $$\tan\theta_{1}=\frac{2t}{1-t^{2}}.$$

Let $\tan\theta_{1}=k$, from which we can derive:

(18) $$kt^{2}+2t-k=0.$$

Since tan*θ*_1_/2 and tan*θ*_1_ have the same sign, *t* and *k* also have the same sign. Solving Equation (18) yields:

(19) $$t=\frac{-1+\sqrt{1+k^{2}}}{k},$$

Given that $\tan\theta_{1}=k$, substituting Equations (12) and (19) into Equation (16), and after algebraic simplification, we obtain:

(20) $$\frac{dy_{m}}{dx_{m}}=\frac{\sqrt{{\left(x-x_{m}\right)}^{2}+y^{2}}+\left(x-x_{m}\right)}{y},$$

where *y* represents the longitudinal coordinate (i.e., the height of the incident point) of the incident ray at the reflecting surface, and *x* represents the transverse coordinate of the reflected ray at the image plane. From Equation (11), *x* can be expressed as an explicit function of *y*, and thus can be completely eliminated in subsequent derivations.

In the *xOy* plane, the coordinates of two adjacent sampling points in the longitudinal subregion of the incident Gaussian beam are denoted as *y*_1_ and *y*_2_; their corresponding reflection points on the freeform mirror have the same longitudinal coordinates, i.e., satisfying *y*_m1_ = *y*_1_ and *y*_m2_ = *y*_2_.

The relationship between the coordinates *y*_m1_ and *y*_m2_ of adjacent points can be expressed as:

(21) $$y_{m2}=y_{m1}+\Delta y_{m},$$

where Δ*y*_m_ is a sufficiently small increment to ensure adequate accuracy of the constructed mirror generatrix.

Based on the geometric constraints of the mirror slope at the reflection point, we obtain:

(22) $$x_{m2}=\frac{dx_{m1}}{dy_{m1}}\Delta y_{m}+x_{m1},$$

where (*x*_m1_, *y*_m1_) represents the coordinate of the reflection point on the mirror corresponding to the starting boundary of the incident sub-region; *x*_m1_ is uniquely determined by the relative spatial position between the mirror and the target spot, as well as the law of reflection, and its value is independent of the output intensity distribution *I*_out*,n*_ of the target spot. According to Formulas (21) and (22), (*x*_m2_, *y*_m2_) can be obtained.

Starting from the point (*x*_m2_, *y*_m2_), successive numerical iterations using Formulas (21) and (22) yield the adjacent discrete point (*x*_m3_, *y*_m3_) on the mirror surface. By continuing this recursive process, subsequent coordinate points are generated step by step, ultimately forming a discrete set of generatrix points corresponding to the longitudinal subregion.

For the remaining longitudinal subregions of the incident beam, the same iterative procedure is applied independently to construct their respective mirror generatrices. All adjacent generatrices meet continuity conditions at their connecting points. Subsequently, the set of discrete generatrix points is fitted with a polynomial, and the resulting fitted curve is then translated and stretched along the direction perpendicular to the *xOy* plane, thereby generating an overall three-dimensional freeform mirror surface.

## 3. Simulation and Experiment

### 3.1. Simulation and Analysis

The mirror’s generatrix equation fitted by the method described in this paper is imported into the SolidWorks 2021(SP0) software, where a three-dimensional mirror surface geometry model was created by extruding along the *z*-direction. The model was then exported in STEP format and precisely loaded into Zemax using the “Import CAD” function. A Gaussian beam with a wavelength of 632.8 nm and a beam waist radius of 15 mm was set as the light source. Based on this model and light source parameters, simulation analyses of intensity distributions were conducted for both uniform and M-shaped spot patterns.

#### 3.1.1. Simulation of Uniform Light Spot in the X-Direction

The incident light field is a Gaussian beam with a waist radius of 15 mm, and the beam center is located at *y*_0_ = 115 mm along the *y*-direction. The corresponding normalized intensity distribution expression is:

(23) $$I_{\mathrm{in}}=\exp(\frac{-2{\left(y-115\right)}^{2}}{15^{2}}),$$

The working distance of the freeform mirror is 300 mm; the output beam shaped by this mirror exhibits a uniform intensity distribution in the *x* direction. The spatial width of the target spot in the *x*-direction is 30 mm, meaning the length of its defined interval |*x*_1_ − *x*_0_| = 30 mm. The normalized intensity distribution of the target spot in the *x*-direction is:

(24) $$I_{\mathrm{out}}=1/30.$$

The incident Gaussian beam is equally divided into three subdomains of identical width along the *y*-direction, and the generatrix of the freeform mirror is segmented into three matching subdomains accordingly. Based on the design method proposed in this paper, we calculate the discrete point coordinates of the reflective surface generatrix, and finally construct a complete generatrix profile comprising 301 data points. The spacing (step size) between adjacent data points is 0.1 mm. The polynomial expression obtained by fitting the mirror generatrix is expressed as:

(25) $$y=\sum_{i=0}^{7}a_{i}x^{i},$$

The polynomial coefficients are shown in Table 1. The fitted polynomial was translated by setting its constant term to zero.

**Figure 3:**
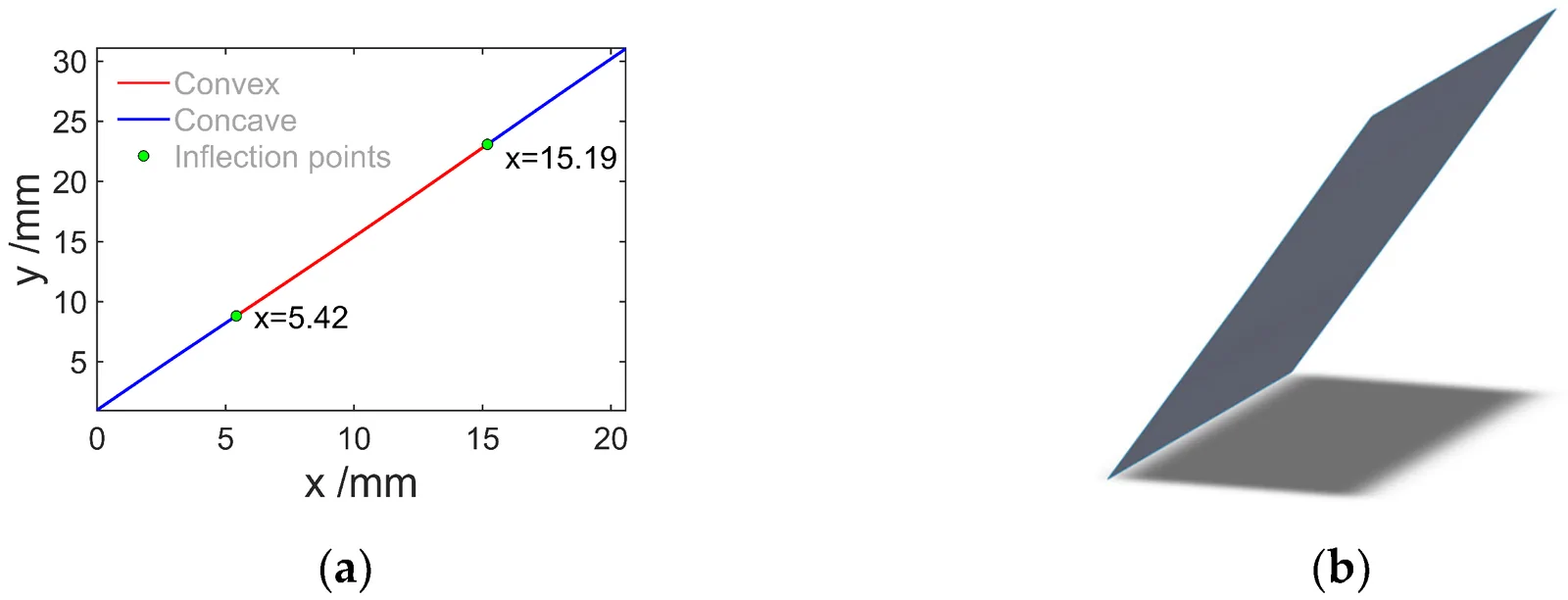
Generatrix and three-dimensional model of a freeform mirror for achieving uniform beam distribution in the *x*-direction: (**a**) the mirror generatrix curve, where the red segment is convex (second derivative greater than zero) and the blue segment is concave (second derivative less than zero); (**b**) the three-dimensional model of the freeform mirror generated by horizontally stretching this generatrix.

As shown in Figure 3a, the generatrix of the freeform mirror exhibits a three-segment geometric profile characterized by “concave–convex–concave.” This structure is designed to match the intensity distribution of the incident Gaussian beam (strong at the center and weak at the edges): the concave regions enhance the convergence of low-intensity edge rays, while the central convex region causes divergence. Combined, these geometric features yield uniform output irradiance along the *x*-direction. The fitted generatrix equation is imported into SolidWorks and extruded horizontally to construct the three-dimensional geometric model of the freeform reflective mirror, as shown in Figure 3b.

**Figure 4:**
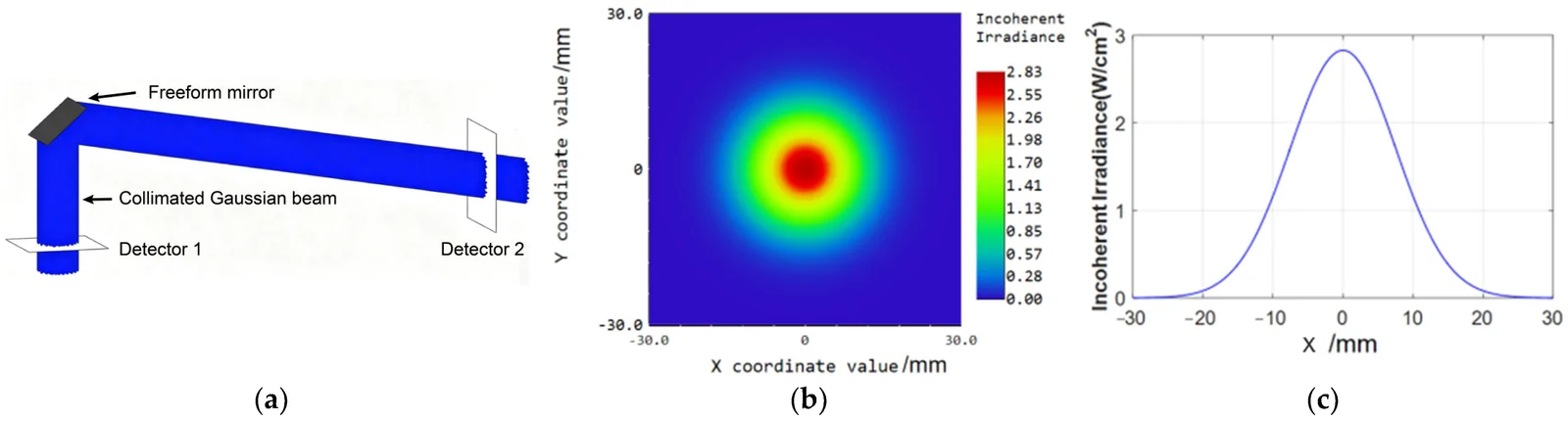
Simulated optical path and incoherent irradiance distribution of Gaussian beam cross-section: (**a**) non-sequential ray tracing diagram of the freeform mirror beam shaping system; (**b**) incoherent irradiance distribution of the incident Gaussian beam in a two-dimensional cross-section; (**c**) incoherent irradiance profile along the *x*-direction in this cross-section.

The surface profile data of the freeform mirror are imported into Zemax for non-sequential ray-tracing simulations (the optical path is illustrated in Figure 4a). In non-sequential mode, the incoherent irradiance distribution of the incident beam is recorded using detector 1. Figure 4b and c respectively depict the two-dimensional cross-sectional incoherent irradiance distribution of the incident Gaussian beam and its intensity profile along the *x*-direction, both of which exhibit excellent agreement with the theoretical Gaussian distribution.

**Figure 5:**
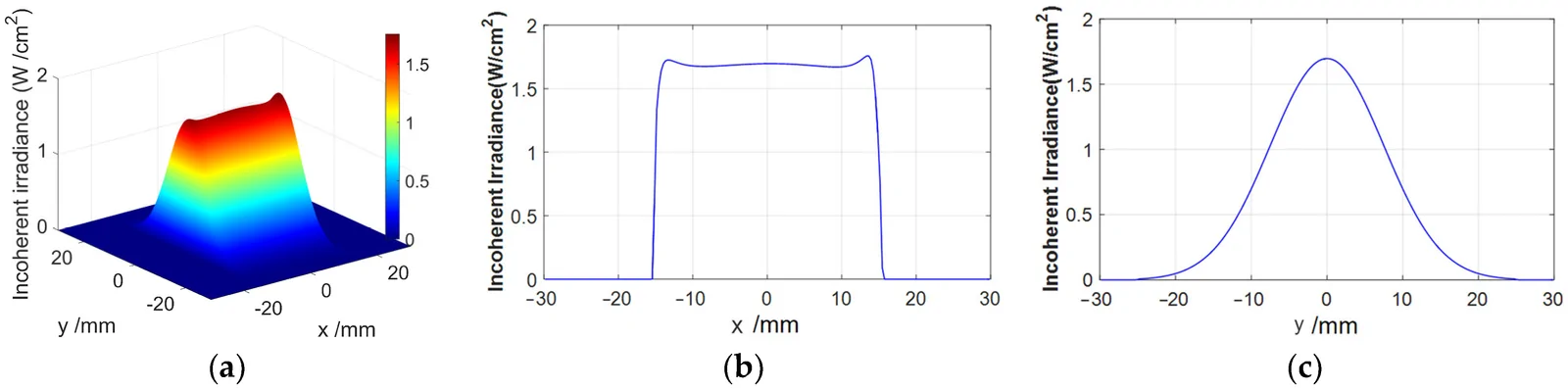
Incoherent irradiance distribution of the shaped Gaussian beam on detector 2: (**a**) three-dimensional intensity distribution of the output spot; (**b**) incoherent irradiance profile in the *x* direction; (**c**) incoherent irradiance profile in the *y* direction.

Intensity data from detector 2 are exported, and the incoherent irradiance distribution of the shaped beam is presented in Figure 5. It can be seen that the incident Gaussian distribution beam in the *x*-direction has been effectively reshaped into an approximately uniform profile.

The quantitative indicators for measuring beam uniformity are uniformity and steepness. According to the international standard (ISO 13694:2018) [18], the beam uniformity is calculated using the following formula:

(26) $$U_{\eta }=\frac{1}{I_{\eta \mathrm{ave}}}\sqrt{\frac{1}{A_{\eta }}\iint {\left[I(x,y)-I_{\eta \mathrm{ave}}\right]}^{2}dxdy},$$

where I(x,y) is the spot intensity, η is the threshold (0<η<1), $I_{\eta \mathrm{ave}}$ is the average value of intensities greater than $\eta I_{\max}$, $A_{\eta }$ is the area where intensity exceeds $\eta I_{\max}$, and $I_{\max}$ is the maximum intensity of the spot. The smaller the uniformity of the spot, the lower the root-mean-square deviation of the intensity from its average, indicating better spot uniformity.

The edge steepness of the target spot is also an important parameter for describing beam shaping performance. According to the international standard (ISO 13694:2018) [18], the edge steepness of the spot is calculated using the following formula:

(27) $$s=\frac{A_{0.1}-A_{0.9}}{A_{0.1}},$$

where $A_{0.1}$ is the area where the light intensity exceeds 0.1 times the maximum intensity, and $A_{0.9}$ is the area where the light intensity exceeds 0.9 times the maximum intensity. *s* approaching zero indicates that the edge of the intensity distribution becomes increasingly vertical.

The output beam spot in the *x*-direction exhibits an intensity uniformity of 4.33% and an edge steepness of 5.88%. In contrast, the *y*-direction retains its original Gaussian distribution characteristics.

It is important to note that the shaping design relies on a freeform surface profile generated via 7th-order B-spline curve fitting in SolidWorks. Insufficient fitting order introduces surface deviations, which subsequently induce local intensity distortions (i.e., “overshoot” and “undershoot” phenomena) at both ends of the *x*-direction. This issue stems from a systematic modeling error rather than simulation or measurement inaccuracies.

#### 3.1.2. Simulation of M-Shaped Light Spot

Reference [11] indicates that a circularly symmetric M-shaped intensity distribution can achieve uniform temperature field within the weld zone during welding. According to the description of M-shaped beams in references [11,19], the peak-to-valley ratio for M-shaped beams is the ratio of the two main side peaks’ maximum intensity to the central valley’s minimum intensity—measured along a chosen direction. To verify the effectiveness of the proposed method and its parameter control capability, we set the target output irradiance distribution $I_{\mathrm{out}}$ to an M-shape and systematically define three sets of significantly different M-shaped spot peak-to-valley ratios, namely 9, 4.5, and 1.5. The mathematical expression for $I_{\mathrm{out}}$ is given below:

(28) $$I_{\mathrm{out}}=\left[1-0.8\cos(\pi x/15)\right]/30,$$

(29) $$I_{\mathrm{out}}=\left[1-0.6364\cos(\pi x/15)\right]/30,$$

(30) $$I_{\mathrm{out}}=\left[1-0.2\cos(\pi x/15)\right]/30,$$

**Figure 6:**
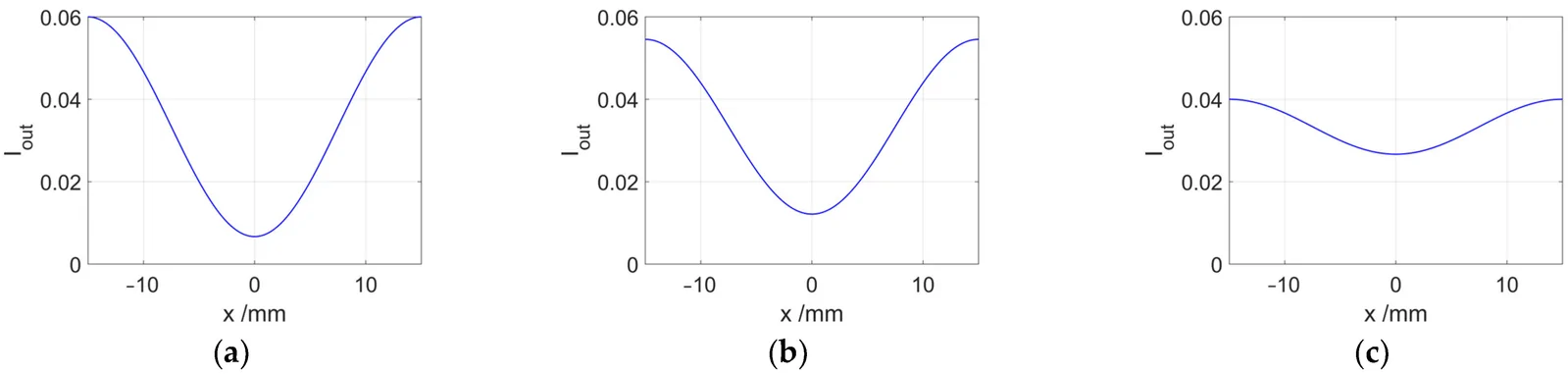
Output M-shaped beam energy distribution curves: (**a**) M-shaped beam energy distribution curve with a peak ratio of 9; (**b**) M-shaped beam energy distribution curve with a peak ratio of 4.5; (**c**) M-shaped beam energy distribution curve with a peak ratio of 1.5.

The irradiance distribution of $I_{\mathrm{out}}$ is presented in Figure 6. The peak-to-valley ratios corresponding to the three M-shaped profiles exhibit a clear decreasing trend.

The beam waist radius and center position of the incident Gaussian beam remain consistent with those in Section 3.1.1. The output intensity distribution $I_{\mathrm{out}}$ adopts three sets of M-shaped intensity functions defined by Equations (28)–(30). Based on the design method proposed in this paper, the generatrix of the mirror is obtained. The polynomial coefficients of the mirror’s generatrix are listed in Table 1.

**Figure 7:**
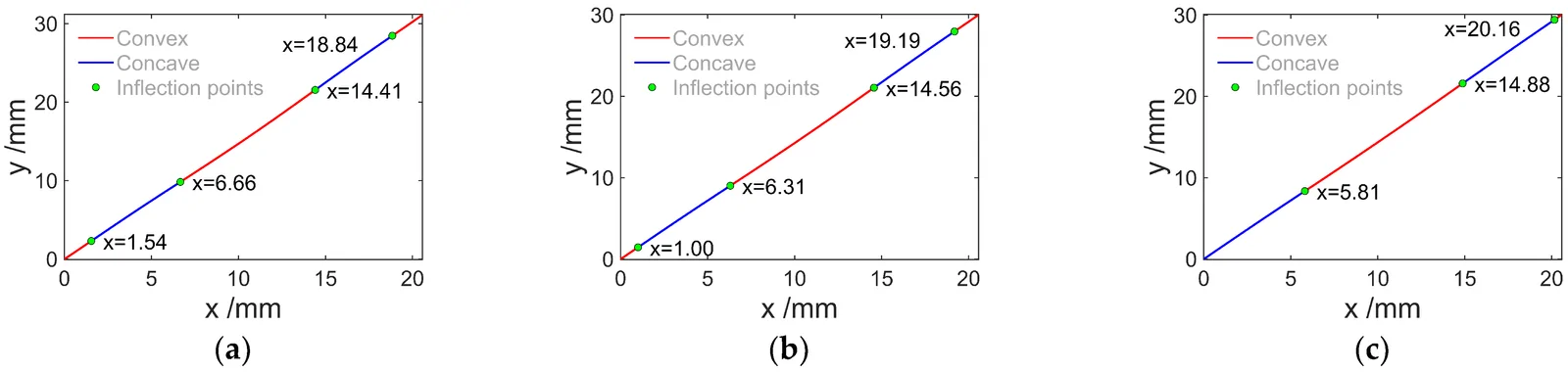
Generatrix of the M-shaped spot mirror surface: (**a**) generatrix of an M-shaped spot mirror surface with a peak ratio of 9; (**b**) generatrix of an M-shaped spot mirror surface with a peak ratio of 4.5; (**c**) generatrix of an M-shaped spot mirror surface with a peak ratio of 1.5.

As shown in Figure 7, the generatrix is divided with convex curves and concave curves alternating. This structure effectively spreads the high energy from the central area of the incident Gaussian beam outward to both sides, while guiding the edge beam to converge moderately, thereby collaboratively forming the required M-shaped light spot.

The generatrix equation was imported into SolidWorks using the same method to construct a freeform surface, then exported for non-sequential ray tracing simulation in Zemax. The optical path configuration used in ray tracing and the intensity distribution of the incident Gaussian beam are both consistent with those shown in Figure 4.

**Figure 8:**
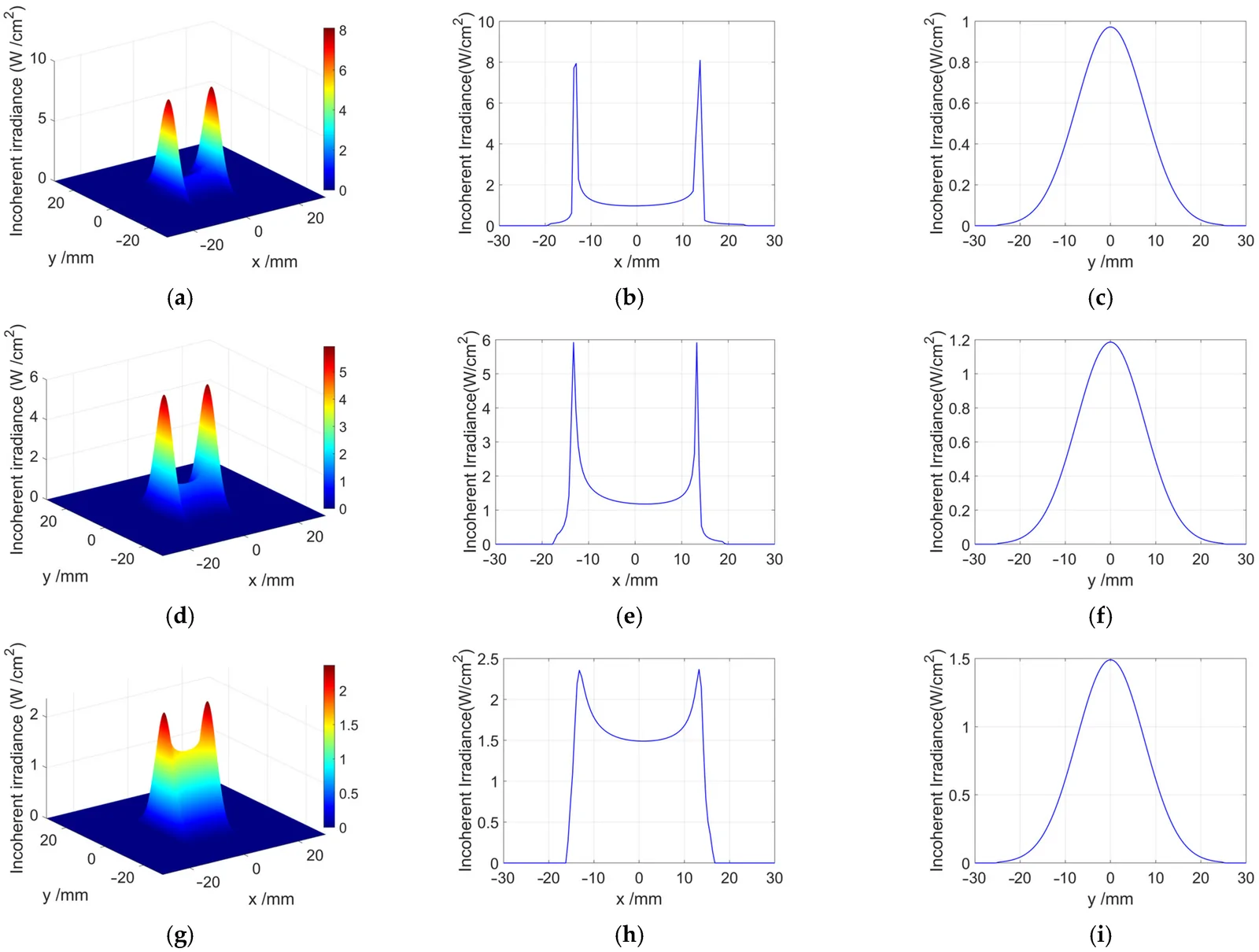
Simulation results of the energy distribution for an M-shaped beam spot: (**a**–**c**) show, respectively, the three-dimensional energy distribution, the cross-sectional energy distribution along the *x*-axis, and the cross-sectional energy distribution along the *y*-axis when the peak-to-valley ratio is 7.95; (**d**–**f**) present the same for a peak-to-valley ratio of 4.96; (**g**–**i**) present the same for a peak-to-valley ratio of 1.56.

Detector 2 is enabled for intensity sampling, and the output beam profiles are illustrated in Figure 8. Along the *x*-axis, the intensity distribution exhibits a distinct double-peak structure. The peak is defined as the maximum intensity at *x* = ±15 mm, and the valley is defined as the intensity value at *x* = 0 mm. The peak-to-valley ratios for the three spots are 7.95, 4.96, and 1.56, respectively, showing a significant stepwise decreasing trend. In contrast, along the *y*-direction, the intensity distribution retains its original Gaussian characteristics without any modulation.

Although fitting errors lead to discrepancies between the design parameters and the actual peak-to-valley ratio of the output spot, this deviation does not affect the overall trend accuracy—the variation pattern of the M-shaped spot’s peak-to-valley ratio is still correctly reflected. This gradient peak-to-valley ratio confirms the effectiveness of the proposed method: by predefining a target M-shaped intensity function, the peak-to-valley ratio of the output beam can be effectively controlled, enabling the generation of M-shaped spots with arbitrary specified peak-to-valley ratios.

### 3.2. Experiment

#### 3.2.1. Freeform Mirror Design and Fabrication

To verify the effectiveness of the proposed design method, an integrator mirror with a diameter of 30 mm was developed based on this method. The working distance (defined as the distance from the integrator mirror to the target spot plane) was set at 300 mm, and the peak-to-valley ratio of the target output beam spot was 1.5. The initial toolpath is generated using a SolidWorks 2026 macro program, followed by tool radius compensation and kinematic correction in the computer-aided manufacturing (CAM) software DIFFSYS 5.1.11.

**Figure 9:**
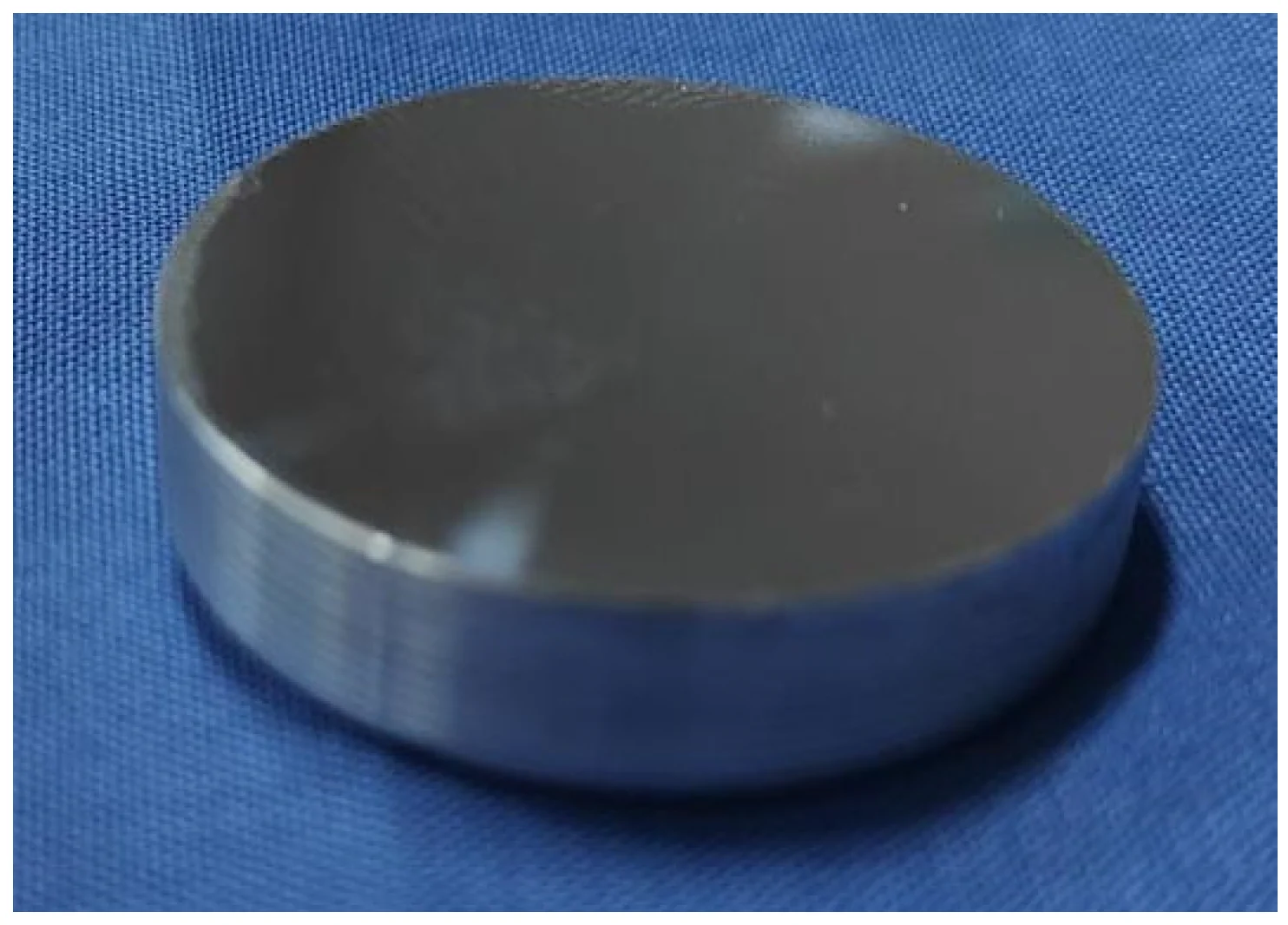
A freeform mirror with a peak-to-valley ratio of 1.5 for the target output beam spot after processing.

The freeform mirror is fabricated from 7075 aerospace Al–Mg alloy and precision-machined on a Moore 250UP ultra-precision single-point diamond turning (SPDT) machine under a constant ambient temperature of 20 ± 0.5 °C. The machining parameters were set as follows: spindle speed = 2000 rpm, feed rate = 5 mm/min, and axial cutting depth = 2 μm. Interferometric measurements (ZYGO) revealed a surface roughness Sa of 12.3 nm across the entire aperture (as shown in Figure 9).

#### 3.2.2. Optical Setup and Characterization

**Figure 10:**
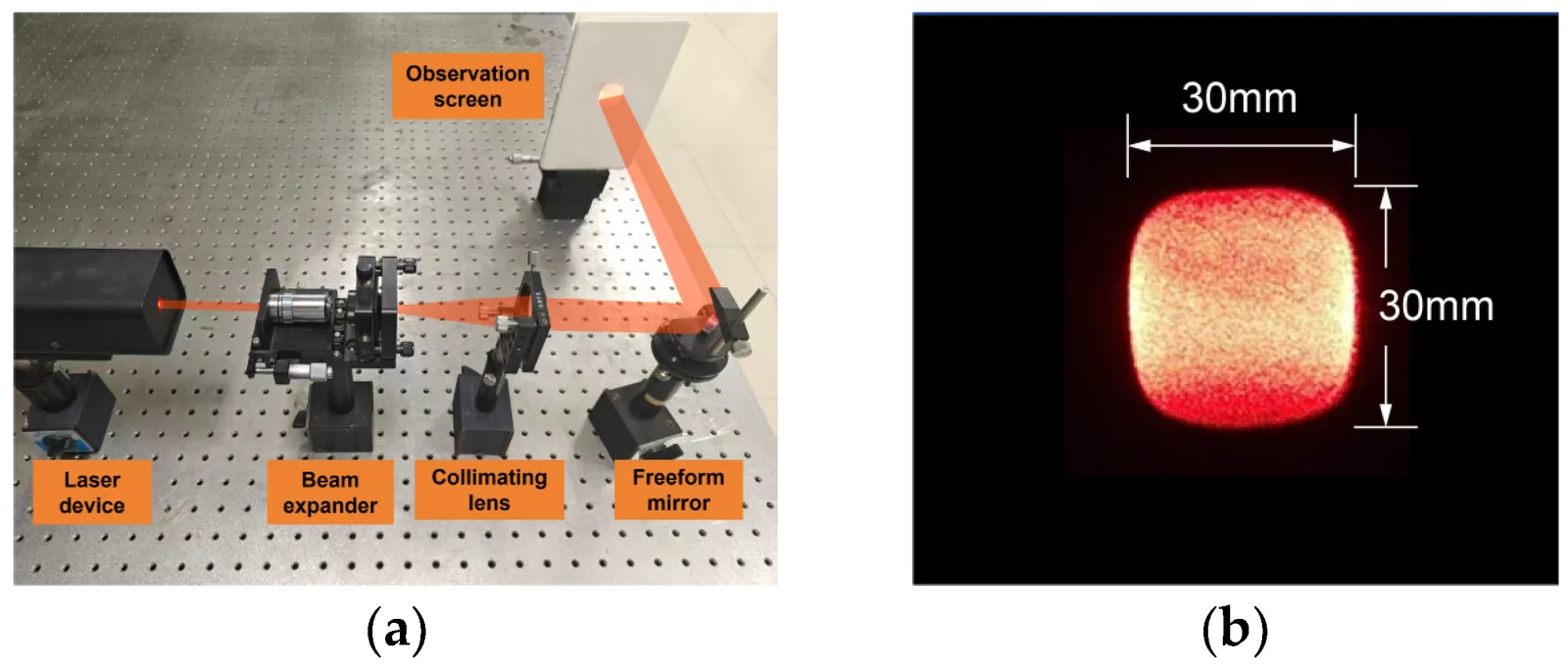
Experimental optical setup and the shaped beam spot: (**a**) experimental optical setup; (**b**) beam spot on the observation screen.

The light source is a helium–neon laser (wavelength λ = 632.8 nm). Its output beam is expanded and collimated to yield a Gaussian beam of 15 mm waist radius, maintaining a Gaussian irradiance distribution prior to incidence on the freeform mirror. The experimental optical path is illustrated in Figure 10a. The collimated Gaussian beam irradiates the freeform reflector at an incident angle of 45°, with a fixed working distance of 300 mm between the mirror and the observation screen.

#### 3.2.3. Results

**Figure 11:**
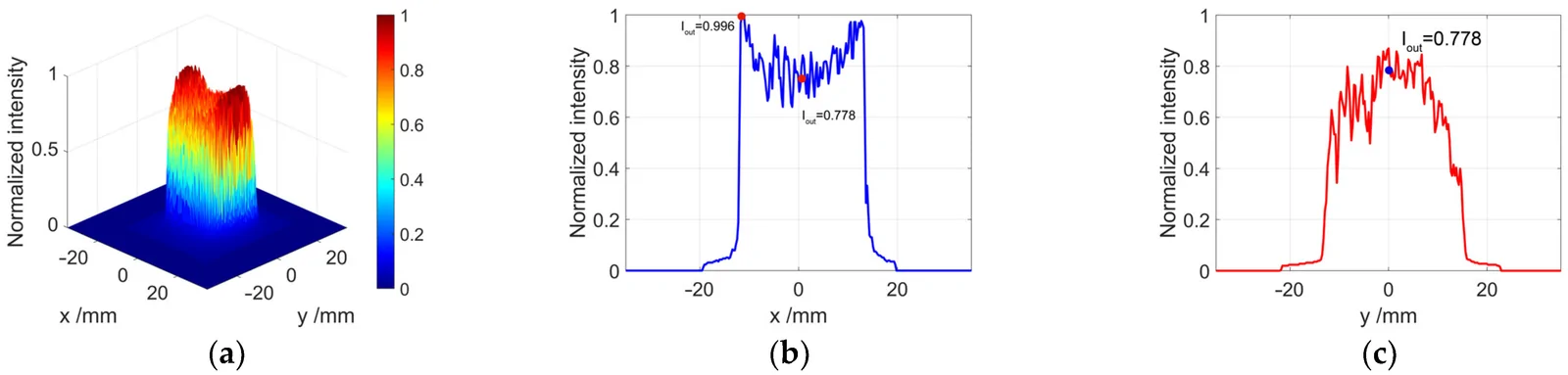
Normalized intensity distribution of the spot on the observation screen experimentally measured: (**a**) three-dimensional normalized intensity distribution of the output spot; (**b**) normalized intensity profile along the *x*-axis; (**c**) normalized intensity profile along the *y*-axis.

The light spot images obtained from the experiment are converted into grayscale images. After baseline correction and linearization processing, the normalized light intensity distribution is calculated. Figure 11a shows the three-dimensional distribution of light intensity on the observation screen; Figure 11b presents the normalized light intensity profile curve along the cross-sectional direction (*x*-axis). The results indicate that the maximum and minimum normalized light intensities on this section are 0.996 and 0.778, respectively, corresponding to a peak–valley ratio of 1.28.

Compared to the simulation results, the peak-to-valley ratio of the measured beam spot in the *x*-direction decreased by 0.28, a reduction of approximately 18%. The maximum deviation between the intensity distribution along the *y*-axis and the ideal Gaussian profile is about 0.1, equivalent to 13% of the maximum intensity value of 0.778 along the *y*-axis.

## 4. Discussion

The difference between the design values and simulation results arises from surface fitting errors. The discrepancies between simulated and experimental results mainly result from the following four types of systematic errors: (1) interpolation error introduced during conversion of freeform surface shape data to the tool path; (2) processing errors caused by machine tool motion inaccuracy, tool wear, and thermal deformation during single-point diamond turning; (3) inherent aberrations of beam-expanding and collimating optical elements (e.g., lenses); and (4) axial and angular misalignment of optical components—such as mirrors and lenses—during optical path alignment. These factors collectively cause deviation between the measured light field and the theoretical expectation.

This study proposes a freeform mirror design that overcomes the limitations of conventional integrator optics—which can generate only uniform beam profiles. The proposed method supports one-dimensional intensity profile control and is extendable to laser cladding, welding, and additive manufacturing. Future work will focus on realizing two-dimensional arbitrary intensity profile generation, integrating high-order NURBS modeling with experimentally measured laser M^2^ values to enhance industrial robustness.

During high-power femtosecond laser processing of metals, beam-induced nonlinear polarization effects significantly influence the energy deposition distribution, ablation threshold, and surface morphology evolution, thereby limiting processing efficiency and quality [20]. It is worth noting that the helium–neon laser used in this experiment has an output power of only 5 mW, aimed at verifying the capability of the proposed M-shaped freeform mirror to achieve the desired peak-to-valley ratio in beam profile at both geometric and optical levels. This experiment serves as a proof-of-concept demonstration rather than a performance evaluation under the actual power levels required for kilowatt-scale industrial laser material processing applications. Future work will focus on experimental validation under high-power conditions. Compare the key process characteristics of flat-top, M-shaped, and ring-shaped laser beams in laser material processing, including energy coupling efficiency, molten pool dynamics, and weld bead formation quality.

## 5. Conclusions

This study proposes an energy-conserving freeform mirror design method for high-power industrial applications, addressing thermal distribution mismatches in laser phase-transformation hardening and cladding processes. The proposed method adopts a zonal energy mapping strategy: it divides the incident Gaussian beam into multiple subregions, independently redistributes irradiance within each subregion to form localized M-shaped distributions with identical peak-to-valley ratios, and finally superimposes them to generate the desired spot profile. Compared to conventional full-aperture energy redistribution approaches, this segmented shaping strategy significantly suppresses error accumulation during numerical inversion, thereby enhancing spatial accuracy and consistency in beam shaping. This design paradigm is particularly suitable for complex industrial scenarios requiring stringent control over irradiance uniformity, thermal flux, and scanning adaptability. Subsequent work will extend this method to generate arbitrary two-dimensional irradiance intensity distributions, supporting the engineering implementation of complex laser heat treatment.

## Figures and Tables

**Table 1:** Polynomial coefficients for fitting the generatrix of a freeform mirror surface.

Polynomial Coefficients	Uniform Distribution in the *x* Direction	M-Shaped Intensity Distribution with a Peak Ratio of 9	M-Shaped Intensity Distribution with a Peak Ratio of 4.5	M-Shaped Intensity Distribution with a Peak Ratio of 1.5
a_0_	0	0	0	0
a_1_	1.474782	1.474782	1.445977	1.466788
a_2_	−7.236737 × 10^−3^	2.4577 × 10^−2^	1.022099 × 10^−2^	−2.208489 × 10^−3^
a_3_	−2.757161 × 10^−6^	−7.069193 × 10^−3^	−4.24821 × 10^−3^	−1.359362 × 10^−3^
a_4_	7.135102 × 10^−5^	5.980609 × 10^−4^	4.409266 × 10^−4^	2.021627 × 10^−4^
a_5_	−3.545972 × 10^−6^	−8.603938 × 10^−6^	−1.329364 × 10^−5^	−8.103409 × 10^−6^
a_6_	1.989075 × 10^−8^	−8.02189 × 10^−7^	−1.558743 × 10^−7^	2.779391 × 10^−8^
a_7_	1.00007 × 10^−9^	2.392583 × 10^−8^	9.453538 × 10^−9^	2.667635 × 10^−9^

## Data Availability

The original contributions presented in this study are included in the article. Further inquiries can be directed to the corresponding authors.
